# Expression and significance of Rac1, Pak1 and Rock1 in gastric carcinoma^2^

**DOI:** 10.1111/ajco.12052

**Published:** 2013-01-08

**Authors:** Yong-jun Wu, Yi Tang, Zhen-feng Li, Zheng Li, Yi Zhao, Zhen-jiang Wu, Qi Su

**Affiliations:** 1 Xiangtan Affiliated Clinical Institute, University of South ChinaXiangtan; 2 University of South ChinaHengyang, China

**Keywords:** gastric neoplasm, immunohistochemistry, Pak1, Rac1, Rock1, tissue microarray

## Abstract

**Aims:**

Rac1, Pak1 and Rock1 are indicators related to gastric cancer invasion and metastasis, but few reports discuss all three kinds of protein in research on gastric cancer invasion and metastasis. The aim of this study was to investigate the expression and clinical significance of Rac1, Pak1 and Rock1 in gastric carcinoma.

**Methods:**

Rac1, Pak1 and Rock1 expression in 158 cases of gastric carcinoma were investigated via immunohistochemical staining and clinical analysis.

**Results:**

The positive expression rates of Rac1, Pak1 and Rock1 in normal tissue, intraepithelial neoplastic tissues and gastric carcinoma showed an increasing trend (*P* < 0.05). Their expression in lymph node metastasis was significantly higher than in patients with lymph-node metastasis than in those without lymph nodes metastasis (*P* < 0.05). Their expression in tumor (TNM stages III and IV) were significantly higher than that in stages I and II (*P* < 0.05). Rac1, Pak1 and Rock1 expression did not differ significantly with patients' sex (*P* > 0.05).

**Conclusion:**

Positive rates of Rac1, Pak1 and Rock1 expression in normal tissue, dysplasia and gastric carcinoma show an increasing trend and are correlated with tumor lymph node metastasis and TNM stage. Rac1, Pak1 and Rock1 may be important biomarkers of gastric carcinoma invasion and metastasis.

## Introduction

Gastric cancer has one of the highest incidences of cancer worldwide. Invasion is a major cause of recurrence and patient death in gastric cancer. Ras-related C3 botulinum toxin substrate 1 (Rac1) is an important member of the small molecule G-protein Rho family (Ras homologue) and is an important class of intracellular signaling molecules. It affects tumor growth, invasion and metastasis, and tumor angiogenesis.^1^,^2^ P21-activated kinase 1 (Pakl) is a conserved serine/threonine protein kinase that is an important downstream target protein of Rho-GTPase Cdc42 and Rac1, which are involved in many important cellular activities and play an important role in cytoskeletal reorganization, cell migration, apoptosis and survival, cell cycle, gene transcription regulation and cell transformation.^3^,^4^ The Rock1 gene is highly expressed abnormally in a variety of tumor cells and plays a role in tumor cell invasion and metastasis.^5^ At present, little research has focused on the relationship between the expression of Rac1, Pak1 and Rock1 in gastric carcinoma and clinical pathology. In the present study, immunohistochemistry and a tissue microarray were used in the detection of Rac1, Pak1 and Rock1 protein expression levels in gastric cancer cells, intraepithelial neoplasia and normal tissues. The correlation between lymph node metastasis and TNM stages was analyzed.

## Methods

### Clinical data

The specimens (resection specimens of 158 cases of gastric cancer) were recruited from the Department of Pathology, Xiangtan Affiliated Clinical College of Nanhua University from 2004 to 2010. All specimens were sorted according to the World Health Organization classification of digestive system cancer in 2010,^6^ and confirmed using hematoxylin–eosin (HE) slice biopsy. The patients had not received radiotherapy and chemotherapy before surgery.

### Tissue microarray

The primary tumor tissue was taken. Based on the slice determined using H–E staining, the representative lesion distribution was determined to construct the tissue microarray.

### Immunohistochemistry

Substance P (SP) immunohistochemistry was carried out according to the manufacturer's instructions. Phosphate buffered saline instead of primary antibodies were used as the negative control. A known cancer-positive biopsy was used as the positive control.

### Results determination

The results were determined according to the method described by Wang *et al*.^7^ The positive rate was determined by three pathology experts. We checked 10 high power fields from each slice randomly, and made a positive cell count score and staining intensity score. Rac1, Pak1 and Rock1 protein positive expression consisted of tan or brown granules located in the cytoplasm and/or cell membrane. Based on the degree of positive staining and the percentage of stained cells, the specimens were scored as follows: 0 corresponds to unstained, 1 point corresponds to brown and 2 points correspond to dark brown; 0 for stained cells <5%, 1 for 5–25%, 2 for 26–50% and 3 for above 50%. Two kinds of scoring were employed: ≥2 points was considered positive, <2 points was considered negative.

### Statistical analysis

The results were analyzed via a χ^2^ test using SPSS 17.0 (SPSS, Chicago, IL, USA) statistical software, and differences of *P* < 0.05 were considered statistically significant.

## Results

### Clinical data

The subjects included 112 men and 46 women with a mean age of 56.25 years (28–83-years old). Gastric cancer with lymph node metastasis was found in 109 cases, and 49 cases did not exhibit lymph node metastasis. A total of 67 cases were classified into TNM stages I to II and 91 were diagnosed with stages III–IV. Intraepithelial neoplastic tissue was collected in 54 cases and normal gastric mucosa (gastric resection specimens from the foci of the cancer more than 10 cm of normal gastric mucosa as the control group) was collected from 64 cases.

### Expression of Rac1, Pak1 and Rock1 protein

**Figure 1:**
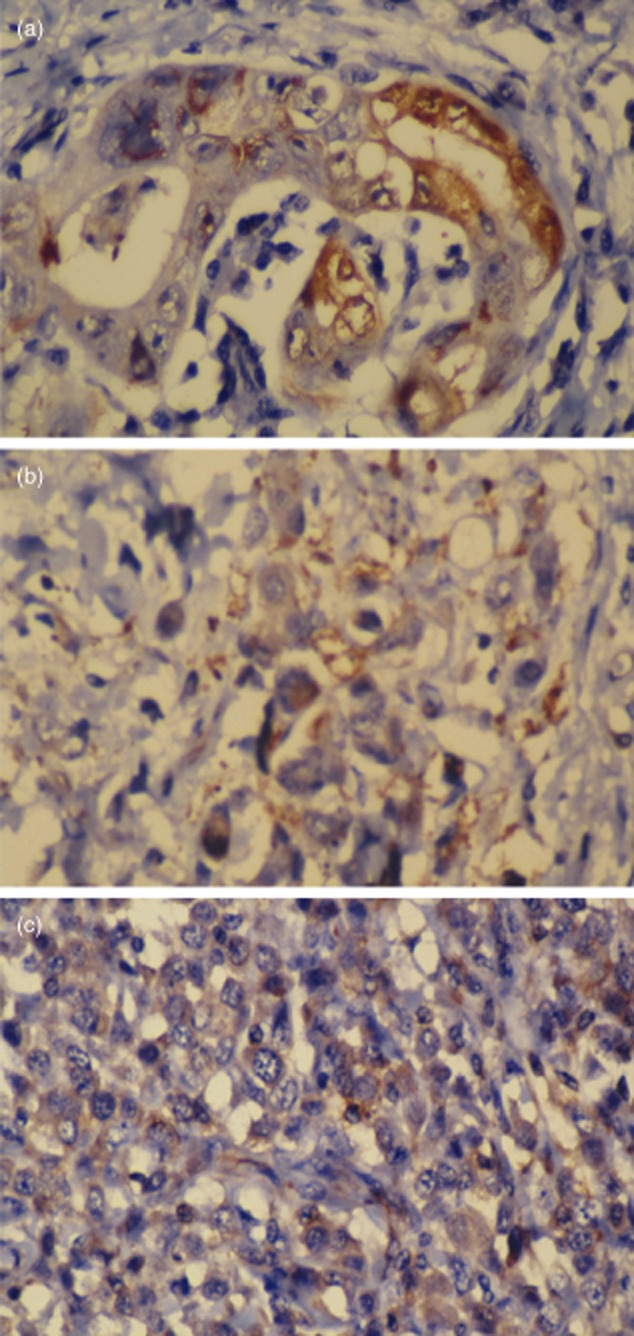
(a) Positive Rac1 in gastric tube adenocarcinoma (substance P (SP) × 400); (b) positive Pak1 in mixed gastric adenocarcinoma (SP × 400); (c) positive Rock1 in stomach cancer of poor adhesion (SP 400).

The expression of Rac1, Pak1 and Rock1 in normal epithelium and intraepithelial neoplastic epithelium was weakly positive or positive; however, a large number of positively stained cells were heterogeneously distributed in the gastric carcinoma tissues. Positive staining was tan-yellow, with bulky granules that were heterogeneously distributed in the cell membrane and cytoplasm. The Rac1 expression rates in the normal gastric tissue, intraepithelial neoplastic tissue and gastric carcinoma were 27, 43 and 68 percent, respectively. The Pak1 expression rates in the three groups were 20, 35 and 60%, respectively, whereas those of Rock1 in the three groups were 16, 28, and 58%, respectively. The differences among the groups were statistically significant (*P* < 0.05) (Fig. 1).

### Relationship between Rac1, Pak1 and Rock1 protein expression and clinicopathological indicators

As shown in Table 1, Rac1, Pak1 and Rock1 expression levels and patients' sex were not statistically significant (*P* > 0.05). Rac1, Pak1 and Rock1 expression in the lymph node metastasis group (75, 71 and 66%) were significantly higher than in the group without metastasis (51, 43 and 41%). Rac1 expression in stages I and II was 48 percent, which was significantly lower than that in stages III and IV (82%). Pak1 expression in stages I and II was 46 percent, which was significantly lower than that in stages III and IV (74%). Rock1 expression in stages I and II was 42 percent, which was significantly lower than that of stages III and IV (70%) (*P* < 0.05).

**Table 1:** Relationship between the expression of Rac1, Pak1 Rock1 and gastric cancer clinical and pathological features

		Rac1		Pak1		Rock1	
				
Clinical pathological features		Positive rate (%)	*P* value	Positive rate (%)	*P* value	Positive rate (%)	*P* value
Sex							
Male	112	73 (65)	0.350	67 (60)	0.586	63 (56)	0.481
Female	46	34 (74)		31 (68)		29 (63)	
Group							
Normal epithelial	64	17 (27)	0.000	13 (20)	0.000	10 (16)	0.000
Epithelial neoplasia	54	23 (43)		19 (35)		15 (28)	
Gastric cancer	158	107 (68)		98 (62)		92 (58)	
Lymph node metastasis							
No	49	25 (51)	0.003	21 (44)	0.001	20 (41)	0.005
Yes	109	82 (75)		77 (71)		72 (66)	
TNM classification							
I, II stage	67	32 (48)	0.000	31 (46)	0.001	28 (49)	0.001
III, IV stage	91	75 (82)		67 (74)		64 (70)	

[Correction added on 19 March 2013, after first online publication: Positive rate (%) of Rac1 for Male was amended to be 65.]

### The relationship between Rac1, Pak1, and Rock1 expression and clinical pathology

The correlation among the expression of Rac1, Pak1 and Rock1 in gastric cancer was analyzed using Spearman's rank correlation. Rac1, Pak1 and Rock1 expression in gastric cancer was positively correlated (r = 0.555, *P* < 0.05).

### Relationship between Rac1, Pak1 and Rock1 expression and survival of gastric cancer patients

**Figure 2:**
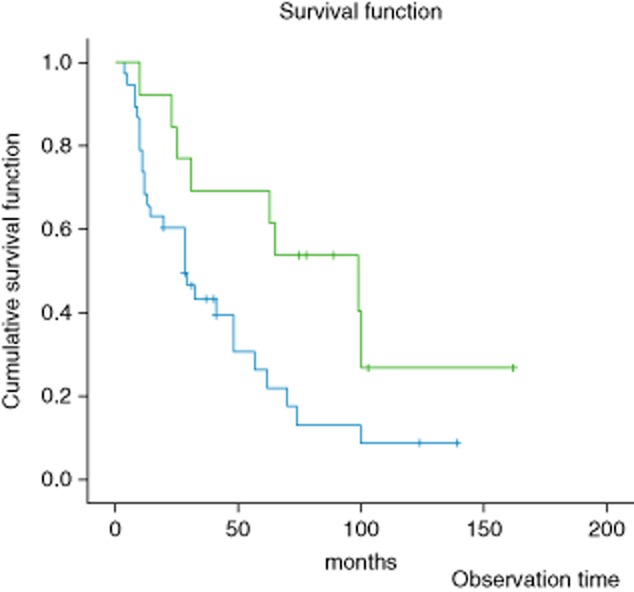
Relationship between Rac1, Pak1 and Rock1 expression and survival of gastric cancer patients showing (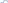) positive, (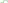) negative, (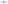) positive deletion and (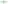) negative deletion groups.

Kaplan–Meier survival curves showed that the median survival time of the 158 patients with follow up was 26 months. The 3-year and 5-year overall survival rates of the whole group were 47 and 41 percent, respectively. Median survival was significantly shorter in Rac1, Pak1 and Rock-positive patients than in Rac1, Pak1 and Rock-negative patients (19 months *vs* 78 months, χ^2^ = 6.857, P = 0.009) (Fig. 2). Univariate survival analysis showed that Rac1, Pak1 and Rock1 expression was a risk factor affecting the survival of the patients.

The results of multivariate analysis by Cox regression for all patients among the four prognostic factors (tumor differentiation, lymph node metastasis, TNM stage and expression of the markers) showed that the expression of the markers may be recognized as the significant independent factor related to disease-free survival (χ^2^ = 17.594, *P* < 0.001).

## Discussion

The characteristics of the cytoskeletal structure result in different exercise capacities, which are related to the genetic diversity of tumor cells and normal cells and different metastatic potentials of the tumor cells.^8^ The cytoskeleton is the intracellular structure mainly composed of protein fiber. It plays an important role in maintaining cell morphology and the internal structure of cells, as well as cell movement, material transport, energy conversion, information transfer and cell differentiation.^9^

The Rac1 gene, located on the short arm of human chromosome 7 (7p22), encodes a small G-protein that is an important member of the Rho (Ras homologue) family. The Rac1 protein has two states, Rac1-GDP (inactive) and Rac1-GTP (activated).^2^ The biological functions of Rac1 depends on its conversion between the two states. When Rac1 is activated it participates in the formation of actin stress fibers and adhesion plaques, promotes cytoskeleton reorganization, regulates sheet pseudopodia and filopodia extension, affects the structure and polarization of the cell, promotes cell motility and migration and inhibits apoptosis.^10^ Studies in recent years have shown that Rac1 expression in colon, breast and lung cancer, among others, was significantly increased. Moreover, Rac1 expression is closely related to invasion and metastasis.^11^,^12^ Rac1 cell motility signaling, which promotes cancer cell invasion and metastasis, is quite complex. This process may be achieved through the following ways: (i) activated Rac1 promotes the assembly of cell surface integrin protein molecules in the surface of the head of the cells and passes regulative signals to the actin cytoskeleton, thus inducing actin filaments to aggregate in the plasma membrane and form sheet pseudopodia, leading to cell membrane multi-polarization, which eventually affects the movement of cell migration'^2^ (ii) Rac1, through the activation of type IV collagenase type 2 matrix metalloproteinase to increase collagenase type I expression, promotes extracellular matrix degradation and enhances the penetration ability of tumor cells.^13^ (iii) Rac1 regulates nuclear factor kappa-light-chain-enhancer of activated B cells activity and increases intracellular superoxide anion concentration to suppress apoptosis.^14^

Pakl is a class of evolutionarily conserved serine/threonine protein kinase that is widely expressed in many tissues as downstream target proteins of the small molecule G-protein Rho family Cdc42 and Rac l. Pakl can be activated by growth factors and other extracellular signals, either through the GTPase-dependent signaling pathway or not. It has a variety of biological effects. PAK, as an important biological regulator, plays an important role in a series of cell functions of mammals, such as cell motility, cell survival, cell cycle, angiogenesis and the regulation of gene transcription. Recent research indicates that Pakl activation of lysophosphatidic acid and toxins from the body induces cell motility in melanoma cells.^15^ Head and neck cancer was found to have higher Pakl activity than normal tissue.^16^ In Kaposi's sarcoma the activity of Pakl is enhanced.^4^ Combined analysis using gene hybridization array and tissue microarray confirmed that Pakl is an upregulated key cancer target gene and it is positively correlated with cyclin D1 expression.^17^ Studies have shown that with increasing Pakl expression, the malignant evolution of colorectal cancer is increased.^18^ Moreover, 55 percent of breast cancer had a high expression of Pakl.^19^

Rock (Rho-associated kinases) are direct downstream target proteins of RhoA, which are involved in a variety of cell functions, such as smooth muscle contraction, cytoskeleton construction, cell adhesion and movement and gene expression. The *Rock* gene has two subtypes, *Rock1* and *Rock2*. *Rock1* is located on chromosome 18 and encodes a 1354 amino acid protein. *Rock2* is located on chromosome 12 and encodes a 1388 amino acid protein. Rocks include an amino acid kinase domain, a carboxyl terminal cysteine-rich region, are coiled coil domains in the middle, which includes the Rho-binding domain. Rock1 and Rock2 have 65 percent amino acid homology and the kinase domain has 92 percent homology. Rock1 is a GTP-dependent serine/threonine protein kinase that interacts with the Rho G-protein through its Rho-binding domain, thereby mediating Rho signaling. Rock1 overexpression or activation stimulates Rho activity. In addition, Rock1 can independently stimulate Rho and directly regulate cell biological behavior. Activated Rock1 induces a variety of proteins that can regulate cytoskeleton phosphorylation, thus producing corresponding biological effects, such as the reliance of Rock1 on MLC kinase or direct phosphorylation of serine 19 of MLC. Through the phosphorylation of LIMK-1 of Section 508 threonine and LIMK-2 of Section 505 threonine and the ezrin-radixin-moesin family proteins and adducin to promote cell cortex actin network formation and actin filament contact with the cell membrane.^17^,^20^

Some studies have shown that GTP enzymes, such as Rho, Rac and Cdc42, through the downstream effectors Pak1, Pak4 and Rock activate LIMK1.^17^,^21^ Previous studies have shown that LIMK1 is closely related to the differentiation of gastric cancer, lymph node metastasis and TNM stage, and plays an important role in invasion and metastasis in gastric cancer. Rac1, Pak1 and Rock1, through the phosphorylation activation of the threonine residue within the LIMK1 ring, regulates the activity of LIMK1 and plays a role in cancer invasion and metastasis.^22^ LIMK is regulated by a variety of upstream signals, where the main upstream signal involved in the migration and invasion is the Rho GTP enzyme family. The Rho GTP enzyme family, including Rho, Rac and Cdc42, are activated by different transmembrane receptors and transmit signals to downstream effector proteins Rock1 and Pak1. Rock1, a Rho-associated protein kinase 1, can activate the protein function, and Rac can indirectly activate LIMK1 by Pak1 (P21-activated protein kinase1). Conformational changes of Rock1 and Pak1 caused by connecting to the active GTP enzyme leads to the first 508 threonine phosphorylation of LIMK1, thereby causing the third serine phosphorylation of cofilin1, ultimately causing actin dynamics,^23^–^28^ caused the formation of a signaling pathway regulation of cell migration and invasion of Rho-Rac1-ROCK1/PAK1-LIMK1-Cofilins. (actin-depolymerizing factor)/cofilins belongs to actin depolymerization factor, is a key factor regulating the actin cytoskeleton. It includes three members: destrin (ADF), cofilin1 and cofilin2, low concentration monomers G-actin, maintaining the actin monomers pool; high concentration by nucleation effect, promoting the formation of pseudopodia, drive tumor cell migration. The results indicate that Rac1 expression in normal gastric tissue, intraepithelial neoplastic tissues and gastric carcinoma were 27, 43 and 68 percent, respectively.

Pak1 expression levels in normal gastric tissue, intraepithelial neoplastic tissues and gastric carcinoma were 20, 35 and 60 percent, respectively, whereas Rock1 expression was 16, 28 and 58 percent, respectively. The difference between the groups was significant (*P* < 0.05). The Rac1 expression rate in lymph node metastasis was 75 percent, which is significantly higher than in the group without metastasis (51%). The Pak1 expression rate in lymph node metastasis was 71 percent, which is significantly higher than in the group without metastasis (43%). The Rock1 expression rate in lymph node metastasis was 66 percent, which is significantly higher than in the group without metastasis (41%) (*P* < 0.05). The Rac1 expression rate in stage II was 47.8 percent, which was significantly lower than that in stage III (82%). The Pak1 expression rate in stage II was 46 percent, which is significantly lower than that in stage III (74%). The Rock1 expression rate in stage II was 42 percent, which is significantly lower than that in stage III (70%) (*P* < 0.05). The correlation of Rac1, Pak1 and Rock1 expression in gastric cancer was analyzed using Spearman's rank correlation, and the expression of the three groups in gastric cancer was positively correlated (r = 0.555, *P* < 0.05). Using a Kaplan–Meier curve to analyze the survival of 158 patients with follow up, it was found that Rac1, Pak1 and Rock expression was closely associated with survival and was a risk factor for survival.

We found that there were significant differences of Rac1, Pak1 and Rock expression in gastric cancer, epithelial neoplasia and normal epithelial tissue, which was an early molecular event in gastric cancer; Rac1, Pak1 and Rock expression, closely related to lymph node metastasis, depth of invasion and degree of differentiation, were valuable indicators in evaluating the degree of malignancy in gastric cancer and thus could contribute to the predication of invasion and metastasis of gastric cancer. Rac1, Pak1 and Rock expression was closely related to the survival of patients and could help in predicting the prognosis of patients. The possible mechanism of interaction between Rac1, Pak1 and Rock and other genes in gastric cancer deserves further study.

Rac1, Pak1 and Rock1 expression in gastric cancer is closely related with the degree of gastric cancer lymph node metastasis and TNM stage and they play an important role in the invasion and metastasis of gastric cancer and might be key biological markers for invasion and metastasis. The expression of these genes might be valuable indicators for evaluating the degree of malignancy of gastric cancer, perhaps as new markers of the biological behavior of gastric cancer. They could contribute to predicting gastric cancer invasion, metastasis and prognosis of patients. The actin cytoskeleton dynamics mechanism in tumor biology behavior, as the regulation of the actin cytoskeleton in cancer prevention and control in the sense Rac1, Pak1 and Rock1 is worth studying. Drugs including paclitaxel and cytochalasin B in making the cytoskeleton stable have been used for the treatment of cancer, but the wide range of their toxic effects make people worry. The development of drugs regulating the expression of Rac1, Rock1 and Pak1 may more accurately regulate actin activity which may be more beneficial in the prevention and treatment of diseases such as cancer.
